# Antimycobacterial and Anti-Inflammatory Activities of Substituted Chalcones Focusing on an Anti-Tuberculosis Dual Treatment Approach

**DOI:** 10.3390/molecules20058072

**Published:** 2015-05-05

**Authors:** Thatiana Lopes Biá Ventura, Sanderson Dias Calixto, Bárbara de Azevedo Abrahim-Vieira, Alessandra Mendonça Teles de Souza, Marcos Vinícius Palmeira Mello, Carlos Rangel Rodrigues, Leandro Soter de Mariz e Miranda, Rodrigo Octavio Mendonça Alves de Souza, Ivana Correa Ramos Leal, Elena B. Lasunskaia, Michelle Frazão Muzitano

**Affiliations:** 1 Laboratório de Biologia do Reconhecer, Centro de Biociências e Biotecnologia, Universidade Estadual do Norte Fluminense Darcy Ribeiro, Campos dos Goytacazes 28013-602, RJ, Brazil; E-Mails: thativentura@yahoo.com.br (T.L.B.V.); sandersoncalixto@yahoo.com.br (S.D.C.); 2 Laboratório de Produtos Bioativos, Curso de Farmácia, Universidade Federal do Rio de Janeiro, Campus Macaé, Pólo Novo Cavaleiro—IMMT, Macaé 27933-378, RJ, Brazil; 3 Faculdade de Farmácia, Universidade Federal do Estado do Rio de Janeiro, Rio de Janeiro 21941-901, RJ, Brazil; E-Mails: babi_abrahim@hotmail.com (B.A.A.-V.); amtsouza2@yahoo.com.br (A.M.T.S.); rangelfarmacia@gmail.com (C.R.R.); ivanafarma@yahoo.com.br (I.C.R.L.); 4 Instituto de Química, Universidade Federal Fluminense, Niterói, Rio de Janeiro 24020141, RJ, Brazil; E-Mail: mvpmello@id.uff.br; 5 Instituto de Química, Universidade Federal do Rio de Janeiro, Rio de Janeiro 21941-909, RJ, Brazil; E-Mails: leandrosoter@iq.ufrj.br (L.S.M.M.); souzarod21@gmail.com (R.O.M.A.S.)

**Keywords:** tuberculosis, *Mycobacterium*, inflammation, chalcone

## Abstract

Tuberculosis (TB) remains a serious public health problem aggravated by the emergence of *M. tuberculosis* (Mtb) strains resistant to multiple drugs (MDR). Delay in TB treatment, common in the MDR-TB cases, can lead to deleterious life-threatening inflammation in susceptible hyper-reactive individuals, encouraging the discovery of new anti-Mtb drugs and the use of adjunctive therapy based on anti-inflammatory interventions. In this study, a series of forty synthetic chalcones was evaluated *in vitro* for their anti-inflammatory and antimycobacterial properties and *in silico* for pharmacokinetic parameters. Seven compounds strongly inhibited NO and PGE_2_ production by LPS-stimulated macrophages through the specific inhibition of iNOS and COX-2 expression, respectively, with compounds **4** and **5** standing out in this respect. Four of the seven most active compounds were able to inhibit production of TNF-α and IL-1β. Chalcones that were not toxic to cultured macrophages were tested for antimycobacterial activity. Eight compounds were able to inhibit growth of the *M. bovis* BCG and Mtb H37Rv strains in bacterial cultures and in infected macrophages. Four of them, including compounds **4** and **5**, were active against a hypervirulent clinical Mtb isolate as well. *In silico* analysis of ADMET properties showed that the evaluated chalcones displayed satisfactory pharmacokinetic parameters. In conclusion, the obtained data demonstrate that at least two of the studied chalcones, compounds **4** and **5**, are promising antimycobacterial and anti-inflammatory agents, especially focusing on an anti-tuberculosis dual treatment approach.

## 1. Introduction

Inflammation is an essential protective response to a variety of noxious stimuli and conditions, such as infection and tissue injury. The inflammatory reaction, characterised by vasodilatation and recruitment of leukocytes into the target tissue, is coordinated by inflammatory mediators produced mainly by macrophages and monocytes, highlighting tumor necrosis factor (TNF), interleukin-1 (IL-1), nitric oxide (NO) and prostaglandins, including PGE_2_ [1,2,3,4].

A potentially beneficial role of inflammation in elimination of invaders could be abrogated by tissue damage in cases of excessive inflammatory response. In chronic infectious diseases, such as tuberculosis, exacerbated inflammation contributes to severe lung pathology, leading to lung tissue necrosis, cavities formation and the promotion of mycobacterial dissemination and transmission. Anti-inflammatory drugs, especially corticosteroids, are currently used for adjunctive therapy in most severe life-threatening forms of tuberculosis, such as meningitis and pericarditis, while antibiotics are used to kill the bacteria [5].

Beneficial effects of adjunctive corticosteroids or non-steroid anti-inflammatory drugs (NSAIDs) in the treatment of the severe forms of pulmonary tuberculosis remain uncertain and need new randomised controlled trials. An increasing body of evidence demonstrates that anti-inflammatory therapy reduces mortality in patients exhibiting the hyperinflammatory phenotype that could be determined by host genetic polymorphisms, increased bacterial virulence or specific comorbid states, such as Tuberculosis-Immune Reconstitution Inflammatory Syndrome (TB-IRIS) in patients with TB and AIDS [6,7,8].

The importance of developing new drugs with dual anti-inflammatory and antimycobacterial, activities is highlighted by the emergence of increasing prevalence of multidrug resistant (MDR) TB and extensively drug-resistant (XDR) TB. Drugs with these properties are currently represented by the anti-leprosy drug clofazimine [9] and they are in short supply for tuberculosis, motivating the design and search for novel agents.

Natural and synthetic flavonoids are known for displaying a broad spectrum of pharmacological activities. Chalcones (1,3-diaryl-2-propene-1-ones) and other biogenetically-related compounds belonging to the flavonoid family are natural substances found in a number of plants or prepared synthetically. They consist of two aromatic rings joined by a three-carbon α,β-unsaturated carbonyl system [10]. Chalcones have been found to exhibit many pharmacological activities, including anti-inflammatory [11,12,13] and anti-tuberculosis [14] activities. The reported anti-inflammatory effects of some chalcones have been associated with suppression of inflammatory mediators, such as NO, TNF-α, IL-1β, cyclooxygenase-2 (COX-2) and inducible oxide nitric synthase (iNOS) [12,13,15]. A number of chalcones were shown to have a high inhibitory activity against *in vitro* growth of laboratory *M. tuberculosis* strains H37Rv when used in low concentrations [14,16,17,18,19] and, additionally, demonstrated low cytotoxicity against human cells in toxicity tests [12]. A combination of these properties in one compound could provide an important advantage for chalcones as potential anti-tuberculosis drugs, but chalcones exhibiting both anti-inflammatory and antimycobacterial properties have not yet been identified.

In this study, we screened a series of substituted chalcones for their anti-inflammatory properties, evaluating immunomodulatory effects on LPS-stimulated macrophages, and for their antimycobacterial properties, evaluating inhibitory effects on bacterial growth of avirulent and virulent mycobacterial strains in culture or in infected macrophages. The active chalcones exhibiting dual activities, were submitted to *in silico* analysis of absorption, distribution, metabolism, excretion and toxicology (ADMET) in order to select promising new drugs. In addition, a structure-activity relationship (SAR) study was performed by modelling to predict molecular descriptors for each studied structure and to gain a better understanding of their antimycobacterial activity.

## 2. Results and Discussion

### 2.1. Results

In this study, we evaluated the anti-inflammatory, antimycobacterial and ADMET properties of a set of forty synthetic substituted chalcones **2**–**41** (Table 1) in comparison to a commercially available unsubstituted chalcone (compound **1**). Initially, compounds **1**–**41** were evaluated for their immunomodulatory activity in LPS-stimulated RAW 264.7 macrophage culture, focusing on NO and TNF-α production levels. For each compound a concentration-response curve was plotted, and the IC_50_ values are reported in Table 2. The obtained results show that chalcones **1**–**41** were more potent inhibitors of NO than TNF-α. More pronounced inhibitory activity against NO was presented by compounds **4**, **5**, **12**, **24**, **28**, **29**, **31**, **33**, **40** and **41**; with IC_50_ values lower than 21 µM. The activity of compound **5** with an IC_50_ of 2.1 ± 0.7 µM was particularly noteworthy.

**Table 1:** Structures of representative unsubstituted chalcone and synthesized chalcones with substituents on the A and B rings. 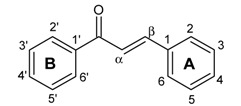

Chalcone	B Ring			A Ring		
	2'	3'	4'	2	3	4
**1**	H	H	H	H	H	H
**2**	H	H	H	H	H	F
**3**	H	H	H	H	H	Cl
**4**	H	H	H	H	H	N(CH_3_)_2_
**5**	H	H	H	H	-O(CH_2_)O-	
**6**	H	H	H	H	H	OCH_3_
**7**	H	H	H	N (pyridine ring)	H	H
**8**	H	H	Br	H	H	H
**9**	H	H	Br	H	H	F
**10**	H	H	Br	H	H	Cl
**11**	H	H	Br	H	H	N(CH_3_)_2_
**12**	H	H	Br	H	-O(CH_2_)O-	
**13**	H	H	Br	H	H	OCH_3_
**14**	H	H	Br	H	OCH_3_	OCH_3_
**15**	H	H	Br	Cl	H	H
**16**	H	H	CH_3_	H	H	H
**17**	H	H	CH_3_	H	H	F
**18**	H	H	CH_3_	H	H	Cl
**19**	H	H	CH_3_	H	H	N(CH_3_)_2_
**20**	H	H	CH_3_	H	-O(CH_2_)O-	
**21**	H	H	CH_3_	H	H	OCH3
**22**	H	H	CH_3_	H	OCH_3_	OCH_3_
**23**	H	H	CH_3_	Cl	H	H
**24**	H	H	OCH_3_	H	H	H
**25**	H	H	OCH_3_	H	H	F
**26**	H	H	OCH_3_	H	H	Cl
**27**	H	H	OCH_3_	H	H	N(CH_3_)_2_
**28**	H	H	OCH_3_	H	-O(CH_2_)O-	
**29**	H	H	OCH_3_	H	H	OCH_3_
**30**	H	H	OCH_3_	Cl	H	H
**31**	Cl	H	H	H	H	H
**32**	Cl	H	H	H	H	F
**33**	Cl	H	H	H	H	Cl
**34**	Cl	H	H	H	H	OCH_3_
**35**	Cl	H	H	H	-O(CH_2_)O-	
**36**	Cl	H	H	H	OCH_3_	OCH_3_
**37**	H	H	H	H	H	NO_2_
**38**	H	H	Br	H	H	NO_2_
**39**	H	H	CH_3_	H	H	NO_2_
**40**	H	H	OCH_3_	H	H	NO_2_
**41**	Cl	H	H	H	H	NO_2_

**Table 2:** Inhibitory effects of chalcones on production of NO and TNF-α by LPS-stimulated RAW 264.7 macrophages, on growth of *M. bovis* BCG in culture and evaluation of chalcone cytotoxicity by LDH test.

Chalcone	IC_50_ (µM)			MIC_50_ (µM)	MIC_90_ (µM)
	NO	TNF-α	LDH	*M. bovis* BCG	
**1**	41.6 ± 2.0	>480.2	177.3 ± 9.5	137.3 ± 4.7	383.2 ± 9.1
**2**	>442.0	>442.0	>442.0	>442.0	>442.0
**3**	31.9 ± 4.6	60.5 ± 4.7	399.7 ± 4.9	20.7 ± 6.4	169.9 ± 5.4
**4**	12.0 ± 2.2	66.9 ± 4.3	348.4 ± 5.1	14.3 ± 5.8	137.3 ± 7.9
**5**	2.1 ± 2.7	48.1 ± 7.6	>396.4	14.5 ± 6.1	75.3 ± 8.4
**6**	>419.7	>419.7	>419.7	255.9 ± 4.8	249.2 ± 6.2
**7**	>477.9	>477.9	>477.9	35.8 ± 7.2	440.8 ± 9.5
**8**	46.4 ± 2.3	66.9 ± 4.5	191.0 ± 5.2	13.4 ± 4.0	33.7 ± 4.6
**9**	>327.7	>327.7	>327.7	35.8 ± 3.9	308.8 ± 5.3
**10**	68.9 ± 4.3	>310.9	113.4 ± 0.5	22.3 ± 6.3	287.8 ± 3.2
**11**	41.3 ± 3.5	>302.8	132.0 ± 2.9	67.8 ± 3.8	242.1 ± 2.6
**12**	19.6 ± 3.3	>302.0	>302.0	84.9 ± 4.7	229.6 ± 2.2
**13**	52.9 ± 3.6	>315.3	>315.3	220.6 ± 3.6	>315.3
**14**	58.9 ± 6.8	>288.0	>288.0	>288.0	>288.0
**15**	36.7+3.8	>311.0	256.0 ± 3.8	13.8 ± 4.4	265.1 ± 4.5
**16**	>449.9	>449.9	>449.9	>449.9	>449.9
**17**	30.8 ± 4.5	>416.2	289.5 ± 4.3	61.5 ± 7.6	388.4 ± 7.3
**18**	150.3 ± 3.8	>389.5	304.3 ± 4.2	71.3 ± 4.8	323.2 ± 7.1
**19**	27.8 ± 3.8	>376.9	>376.9	56.4 ± 4.4	322.7 ± 1.7
**20**	30.1 ± 1.2	>375.5	>375.5	>375.5	>375.5
**21**	30.6 ± 4.7	>396.3	>396.3	33.3 ± 5.4	362.2 ± 5.9
**22**	>354.2	>354.2	>354.2	>354.2	>354.2
**23**	>389.5	>389.5	>389.5	>389.5	>389.5
**24**	21.3 ± 4.5	78.8 ± 6.5	244.8 ± 4.5	31.5 ± 5.9	399.1 ± 6.9
**25**	41.2 ± 4.6	>390.2	>390.2	15.8 ± 5.2	228.3 ± 5.8
**26**	25.3 ± 3.9	>366.7	>366.7	91.2 ± 5.0	286.7 ± 7.8
**27**	44.6 ± 5.2	>355.4	226.4 ± 3.5	308.8 ± 5.0	>355.4
**28**	14.9 ± 4.1	>354.2	>354.2	>354.2	>354.2
**29**	13.1 ± 4.2	>372.7	93.9 ± 4.9	32.5 ± 5.4	339.5 ± 3.2
**30**	23.9 ± 4.8	>366.7	100.0 ± 5.1	37.8 ± 6.4	328.7 ± 3.6
**31**	8.4 ± 6.8	>412,0	267.0 ± 4.5	90.9 ± 5.5	330.2 ± 4.2
**32**	>383.6	>383.6	>383.6	>383.6	>383.6
**33**	3.1 ± 0.1	165.5 ± 5.2	351.2 ± 5.0	45.7 ± 5.4	>360.8
**34**	>366.7	>366.7	>366.7	>366.7	>366.7
**35**	160.3 ± 4.7	>348.8	>348.8	>348.8	>348.8
**36**	187.8 ± 4.1	>330.3	>330.3	269.6 ± 3.9	>330.3
**37**	58.6 ± 4.5	>394.9	>394.9	>394.9	>394.9
**38**	>301.1	>301.1	>301.1	97.4 ± 4.2	220.7 ± 5.6
**39**	113.7 ± 4.8	>373.2	328.6 ± 3.9	28.1 ± 5.4	344.1 ± 6.3
**40**	13.5 ± 5.5	>353.0	>353.0	14.5 ± 3.1	>353.0
**41**	11.2 ± 5.0	68.54 ± 3.3	>347.6	30.5 ± 4.7	246.9 ± 7.2
**L-*N*MMA** ^1^	78.3 ± 6.5	XX	XX	XX	XX
**Rifampicin** ^2^	XX	XX	XX	0.01 ± 0.03	0.2 ± 0.01

^1^ Standard nitric oxide inhibitor; ^2^ Standard antimycobacterial drug; Mean value ± SD; n = 3; XX—not defined.

It should be noted that the inhibitory activities of the substituted chalcone compounds more active against NO were higher than those of the unsubstituted chalcone (IC_50_ of 41.6 ± 2.0 µM) or L-NMMA, which is a competitive NO inhibitor used as a positive control (IC_50_ of 78.3 ± 6.5 µM). Some of these chalcones (compounds **4**, **5**, **24**, **41**) were able to strongly inhibit TNF-α as well (Table 2). Compound **5** was notably the most potent inhibitor of TNF-α and NO production (*p* < 0.05) when compared to other compounds.

In order to investigate the cytotoxicity of the studied compounds, the levels of intracellular LDH, released in culture supernatants by chalcone-treated macrophages were measured. Compounds **10**, **11**, **29** and **30** showed the worst cytotoxicity profiles (Table 2) and were excluded from further analyses.

As a part of our initial screening strategy, the compounds **1**–**41** were assessed for their antimycobacterial activity in *Mycobacterium bovis* BCG culture. The vaccine strain was used for the screening experiments considering biosecurity reasons. Growth inhibition of mycobacteria cultured in the presence of chalcones was quantified and results are presented in Table 2 as MIC_50_ and MIC_90_. The most potent inhibitory effect was obtained using compounds **5** and **8** that showed the lowest MIC_50_ and MIC_90_ levels (14.5 ± 6.1 and 75.3 ± 8.4 µM for **5**; 13.4 ± 4.0 and 33.7 ± 4.6 µM for **8**). It should be noted that none of the tested compounds was more active than rifampicin, a standard anti-TB drug used as a positive control.

**Figure 1:**
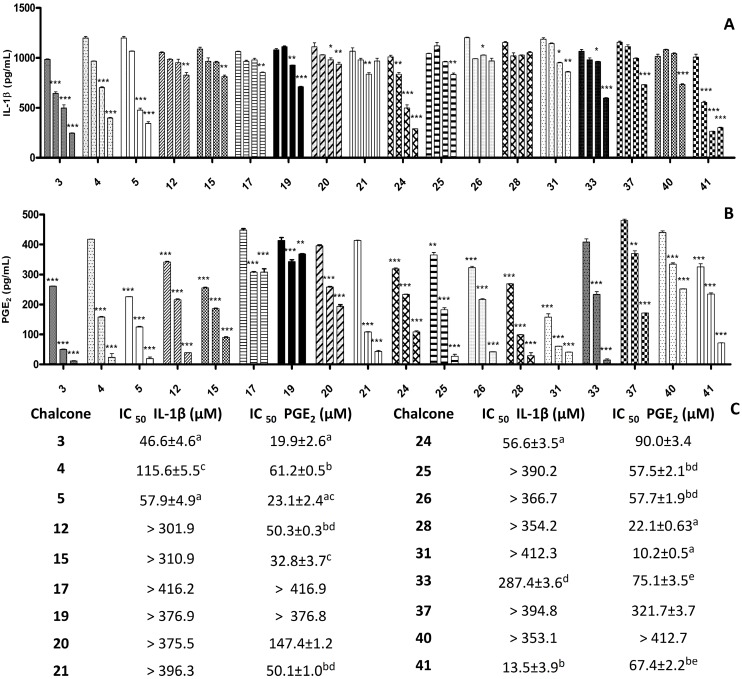
Effect of substituted chalcones on production of proinflammatory mediators (IL-1β and PGE_2_) by LPS-stimulated RAW 264.7 macrophages. RAW 264.7 cells were treated with LPS (1 µg/mL) in the presence of chalcones (0.8, 4, 20 e 100 µg/mL, the lowest concentration was not used in the PGE assay) for 24 h. The culture supernatants were collected and tested by sandwich ELISA for IL-1β (**A**) and PGE_2_ (**B**). In the IL-1β test, cytokine values determined in untreated and only LPS-treated cell cultures were 0.03 ± 0.1 pg/mL and 922.0 ± 4.6 pg/mL, respectively (A). In the PGE_2_ test, prostaglandin values determined in untreated and only LPS-treated cell cultures were 0.05 ± 0.1 pg/mL and 436.0 ± 5.8 pg/mL, respectively. Standard drug, indomethacin was used as positive control, at concentrations of 0.2, 0.04, 0.004 and 0.0004 µg/mL and prostaglandin values obtained were 11.8 ± 1.9; 171.8 ± 1.2; 271.6 ± 2.6 and 340.7 ± 1.1 respectively (B). The bars for each chalcone refer to concentrations tested in ascending order. (**C**) Minimum inhibitory concentrations of substituted chalcones on production of IL-1β and PGE_2_. Statistical analyses were calculated and values in the same column with different superscript letters (a–e) are significantly different (*p* < 0.05 or *p* < 0.001); determined in Tukey test. Values are reported as means ± S.E.M., and differences between groups were considered to be significant at *p* < 0.001 (***), *p* < 0.01 (**) and *p* < 0.05 (*).

Additionally, we evaluated the capacity of chalcones to inhibit production of IL-1β and PGE_2,_ induced in macrophages by LPS. For this study, we selected eighteen chalcones that had shown high activity in the NO inhibition test and were less cytotoxic than compound **1** (a non-substituted chalcone used as a reference compound). Figure 1 shows that inhibitory activity of these chalcones against IL-1β production was relatively low. Only five of these compounds (compounds **3**, **4**, **5**, **24** and **41**) were potent inhibitors of IL-1β. In contrast, the inhibitory activity of most compounds against PGE_2_ was high, with the activity profiles of compounds **3**, **4**, **5**, **28** and **31** standing out.

**Figure 2:**
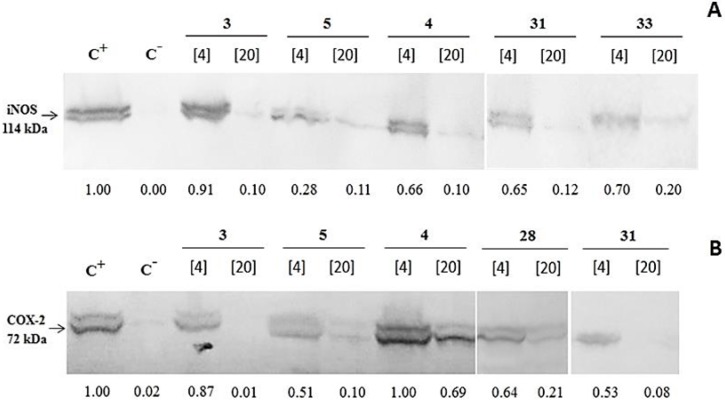
Effects of chalcones on iNOS and COX-2 expression following RAW 264.7 macrophage stimulation by LPS. Cells were treated with LPS (1 µg/mL) and chalcones (4 and 20 µg/mL) for 24 h, lysed and submitted to western blot for analysis of iNOS (**A**) and COX-2 (**B**) expression. The presented results refer to chalcone compounds demonstrating the highest levels of inhibitory activity levels (samples 3, 4, 5, 28, 31, 33). The cells treated with LPS only were used as a positive control of macrophage stimulation (C^+^). Untreated cells were used as a negative control (C^−^). Images are representative of two independent experiments that showed similar results. Lower panel, quantification of the protein levels by immunoreactive bands densitometric analysis. Each band was compared to respective positive control band at same membrane. Relative densities were calculated employing ImageJ software. Densitometric protein bands analysis was performed using ImageJ software for Windows (NIH, Bethesda, MD, USA). The value for positive control condition (LPS-stimulated cells) was set as 1 and other conditions were recalculated correspondingly to allow ratio comparisons.

Seeking to learn more about the mechanism of NO and PGE_2_ inhibition by chalcones, we studied the expression of iNOS and COX-2 in macrophages treated with LPS and chalcone compounds exhibiting the strongest inhibitory activities against NO and PGE_2,_ respectively. As can be seen in Figure 2, compounds **3**, **5**, **4**, **31** and **33**, when used at a concentration of 20 µg/mL, were able to almost completely inhibit iNOS. Some of these chalcones (compounds **3**, **5** and **31**) were most potent in abrogation of COX-2 as well. These results demonstrate that the inhibitory effects of chalcones on NO and PGE_2_ production are mediated by suppression of iNOS and COX-2 expression, respectively.

The second aim of this work was to investigate the antimycobacterial activities of chalcones against pathogenic *M. tuberculosis*. The chalcone compounds presenting inhibitory activities in the screening test for BCG growth inhibition (Table 2) were selected for the further study against laboratory *M. tuberculosi*s strain H37Rv and a highly virulent *M. tuberculosis* clinical isolate, such as strain M299 belonging to the modern *M. tuberculosis* Beijing sublineage [20]. Sixteen compounds were assessed for the inhibitory activity against *M. tuberculosis* H37Rv. The results presented in Table 3 show that most compounds were active against this strain, presenting MIC_50_ values similar to those used against BCG (Table 2), despite a decreasing activity when analyzing MIC_90_ values. It should be noted that the compounds most active against BCG, **5** and **8** (MIC_90_ 75.3 ± 8.4 μM and 33.7 ± 4.6 μM, respectively), were less potent against the virulent H37Rv strain (MIC_90_ 303.2 ± 5.4 μM and 344.7 ± 0.4 μM, respectively).

**Figure 3:**
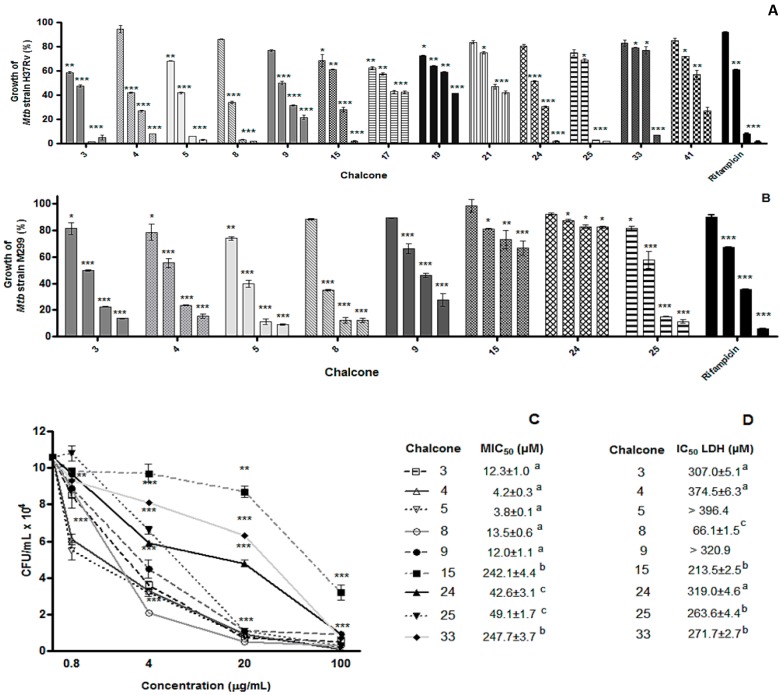
Effect of chalcones on *M. tuberculosis* growth in bacterial culture and macrophages. Bacterial suspensions (1 × 10^6^ CFU/well) of *M. tuberculosis* strain H37Rv (**A**) and clinical isolate M299 (**B**) were treated or untreated with chalcone samples (0.8, 4, 20 and 100 µg/mL) or rifampicin (0.00032, 0.0016, 0.008, 1 µg/mL for the strain H37Rv and 0.008, 0.04, 0.2 and 10 µg/mL for the strain M299) for 5 days. Bacterial growth in the resulted cultures was quantified by MTT test. Data are presented as a percentage of bacterial growth of each treated culture compared to the growth of corresponding untreated culture (100%). The four bars for each compound refer to concentrations tested in ascending order. Bacterial suspensions treated with antibiotic rifampicin were used as a positive control. Untreated bacterial suspension served as a negative control. (**C**, **D**) RAW 264.7 macrophages were infected with *M. tuberculosis* strain H37Rv at the infection ratio of 1:1 (macrophage:mycobacteria) and treated with chalcones for 4 days. Bacterial intracellular viability after the treatment was determined by bacterial CFU counts (C) and toxicity for macrophages was evaluated by LDH assay (D). The results presented are mean values obtained over three experiments, each done in triplicate. *** *p* < 0.001, ** *p* < 0.01 and * *p* < 0.05 compared to untreated group. Values in the same column with different superscript letters (a–c) are significantly different (*p* < 0.05 or *p* < 0.001; determined by Tukey test).

Eight chalcones exhibiting higher levels of antimycobacterial activity were additionally tested against the hypervirulent *M. tuberculosis* strain M299. Only five of them (compounds **3**, **4**, **5**, **8** and **25**) showed similar activity profiles against both *M. tuberculosis* strains (Table 3 and Figure 3), whereas two chalcones (compounds **15** and **24**) were significantly less potent against the clinical isolate M299. It should be noted that the strain M299 was more resistant to the antimycobacterial action of rifampicin as well. Comparable levels of bacterial growth inhibition for these strains were obtained after at least a ten-fold increase in rifampicin concentrations used for the treatment of strain M299 compared with those used for the strain H37Rv (Figure 3A,B).

Additionally, we evaluated the effects of chalcones on intracellular growth of *M. tuberculosis* strain H37Rv in macrophages. The results presented in Figure 3C show that eight of nine studied chalcones, used at a concentration of 100 µg/mL, and six, used at a concentration of 20 µg/mL, were able to reduce drastically (95%–100% inhibition) the intracellular bacillary load demonstrating a good efficacy profile.

**Table 3:** Minimum inhibitory concentrations of substituted chalcones suppressing growth of the laboratory *M. tuberculosis* strain H37Rv and clinical *M. tuberculosis* isolate M299.

Chalcone	MIC_50_ (µM)	MIC_90_ (µM)	MIC_50_ (µM)	MIC_90_ (µM)
	*M. tuberculosis* H37Rv		*M. tuberculosis* M299	
**3**	13.0 ± 3.3 ^a^	372.7 ± 3.6 ^a^	18.37 ± 3.9 ^a^	373.9 ± 1.4
**4**	19.7 ± 1.9 ^a^	320.1 ± 4.8 ^b^	20.1 ± 1.0 ^a^	351.5 ± 1.8
**5**	12.7 ± 1.9 ^a^	303.2 ± 5.4	11.7 ± 3.4 ^a^	289.6 ± 0.7
**7**	>477.9	>477.9	XX	XX
**8**	10.5 ± 3.8 ^a^	325.9 ± 3.3 ^b^	10.2 ± 4.9 ^a^	344.7 ± 0.4
**9**	24.4 ± 4.5 ^a^	252.1 ± 2.7 ^c^	53.58 ± 1.9	313.0 ± 1.4
**15**	17.1 ± 3.8 ^a^	287.8 ± 5.5 ^c^	>311.0	>311.0
**17**	63.8 ± 6.2 ^b^	>416.2	XX	XX
**19**	112.4 ± 6.4 ^c^	>376.8	XX	XX
**21**	66.9 ± 6.2 ^b^	368.2 ± 5.2 ^a^	XX	XX
**24**	25.3 ± 5.2 ^a^	374.6 ± 6.8 ^a^	>419.7	>419.7
**25**	25.1 ± 5.2 ^a^	366.1 ± 3.6 ^a^	18.5 ± 3.6 ^a^	366.9 ± 0.3
**33**	36.9 ± 4.5 ^a^	355.8 ± 4.9 ^a^	XX	XX
**39**	>373.2	>373.2	XX	XX
**40**	131.7 ± 1.3 ^c^	351.8 ± 5.9 ^a^	XX	XX
**41**	82.1 ± 4.3 ^b^	275.3 ± 6.7 ^c^	XX	XX
Rifampicin	0.11 ± 0.02	0.15 ± 0.08	0.2 ± 0.18	3.3 ± 0.16

Values in the same column with different superscript letters (a–c) are significantly different (*p* < 0.05 or *p* < 0.001, determined by Tukey test). XX—not defined.

Four compounds displayed 70%–75% inhibition, when used at a low concentration of 4 µg/mL. The most active compounds **4** and **5** showed the lowest MIC_50_ values, 4.2 ± 0.3 and 3.8 ± 0.1 µM, respectively (Figure 3C). Importantly, the toxic effects of compounds **4** and **5** were selective for mycobacteria, whereas the cytotoxicity measured by LDH release test was low (Figure 3D), demonstrating that these compounds are good candidates for the *in vivo* pharmacological study.

For the further investigation of the pharmacological potential of chalcones, in this study, we performed theoretical calculations aimed at investigating the stereoelectronic properties of the studied substituted and unsubstituted chalcones. Since the number of molecules was not enough for a QSAR investigation, the SAR studies were carried out, to identify physicochemical properties that might be related to antimycobacterial activity.

An overall 2D and 3D-analysis of chemical structures of these derivatives showed electron-withdrawing groups presence substituted at aromatic rings induced an enhancement in the activity. Besides, methyl substituent at *para* ring B position (**17**, **19**, **21** and **39**) seems to decrease antimycobacterial activity.

Analysis of these derivatives electronic properties, including HOMO and LUMO energy and distribution, dipole moment and vector and molecular electrostatic potential maps (MEP), showed that chalcone derivatives present different parameters values, and MEPs alone did not suggest any direct correlation with the antimycobacterial activity (data not shown).

However, the analysis of α,β-unsaturated carbonyl atoms electrostatic charge and dihedral angles showed some correlation with biological activity. It was observed that the less negative oxygen (O1) and less positive carbonyl carbon (C7) result in more active chalcone derivatives (Table 4). It was also observed that a more negative electrostatic charge on the C9 unsaturated carbon causes activity decrease or loss (Table 4).

**Table 4:** α,β-Unsaturated carbonyl chalcones derivatives (O1, C7, C8 and C9) electrostatic charging system distribution and variation in dihedral angles according to the substituent change on aromatic rings. 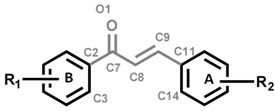

#	O1	C7	C8	C9	C8, C7, C2, C3	C8, C9, C11, C14
**3**	−0.591	0.583	−0.307	−0.169	38.51	−48.75
**4**	−0.608	0.645	−0.434	−0.079	31.08	27.78
**5**	−0.591	0.564	−0.405	−0.036	−38.86	46.97
**8**	−0.589	0.610	−0.364	−0.184	39.72	−47.79
**9**	−0.586	0.582	−0.362	−0.142	39.81	−47.43
**15**	−0.581	0.562	−0.314	−0.116	39.27	−54.30
**17**	−0.606	0.633	−0.349	−0.129	38.56	−47.76
**19**	−0.617	0.646	−0.385	−0.113	41.46	−39.66
**21**	−0.614	0.646	−0.377	−0.103	40.34	−43.67
**24**	−0.613	0.651	−0.32	−0.195	36.6	−48.40
**25**	−0.613	0.650	−0.341	−0.148	36.55	−48.18
**33**	−0.593	0.654	−0.374	−0.124	53.44	−45.43
**40**	−0.603	0.66	−0.289	−0.233	35.21	−49.64
**41**	−0.585	0.659	−0.319	−0.199	−48.82	48.58

Furthermore, it was found that substitution on aromatic rings induces a change in dihedral angle. This may suggest that conformational aspects are influencing these compounds interaction with the *Mycobacterium* macromolecular target.

In addition, we also evaluated Lipinski “Rule of Five”, which is related to oral bioavailability. All active compounds fulfilled Lipinski “Rule of Five” (cLogP ≤ 5, molecular weight ≤ 500, number of hydrogen bond donors ≤ 5 and number of hydrogen-bond acceptors ≤ 10) [21] pointing to a good theoretical oral bioavailability (Table 5).

When any drug candidate is developed, some concern is expected due to the possible lack of adequate ADMET properties, which constitute the principal cause of drug disapproval [22]. Here we evaluated *in silico* the ADMET properties of chalcones active against *M. tuberculosis* strains which showed good *in silico* pharmacokinetic profile. ADMET risk provides a range between 0–24, where greater the number, higher the drug probability of not be well tolerated. ADMET risk results were considered acceptable, since values were between 1 and 3 (data not shown), indicating that all chalcones tested probably do not have ADMET problems.

**Table 5:** Lipinski’s “rules of five” analysis parameters of biologically active chalcone samples.

Chalcone	MIC_50_ (µM)	MW	LogP	HBD	HBA
**3**	13.0 ± 3.3 ^a^	242.70	4.32	0	1
**4**	19.7 ± 1.9 ^a^	251.33	4.04	0	2
**5**	12.7 ± 1.9 ^a^	252.27	3.54	0	3
**8**	10.5 ± 3.8 ^a^	287.16	4.59	0	1
**9**	24.4 ± 4.5 ^a^	305.15	4.75	0	1
**15**	17.1 ± 3.8 ^a^	321.60	5.15	0	1
**17**	63.8 ± 6.2 ^b^	240.28	4.4	0	1
**19**	112.4 ± 6.4 ^c^	265.36	4.53	0	2
**21**	66.9 ± 6.2 ^b^	252.31	4.12	0	2
**24**	25.3 ± 5.2 ^a^	238.29	3.63	0	2
**25**	25.1 ± 5.2 ^a^	256.28	3.79	0	2
**33**	36.9 ± 4.5 ^a^	277.14	4.88	0	1
**40**	131.7 ± 1.3 ^c^	283.28	3.67	0	5
**41**	82.1 ± 4.3 ^b^	287.70	4.35	0	4

Abbreviations: lipophilicity (Log P), molecular weight (MW), number of hydrogen bond donor groups (HBD) and number of hydrogen bond acceptor groups (HBA). Values in the same column with different superscript letters (a–c) are significantly different (*p* < 0.05 or *p* < 0.001; determined in Tukey test).

Furthermore, specific toxicity analysis *in silico* was performed. The biomarkers used for predicted hepatotoxicity diagnostics were alkaline phosphatase (ALP), serum glutamic oxaloacetic transaminase (SGOT), serum glutamic pyruvic transaminase (SGPT), γ-glutamyl transferase (GGT) and lactate dehydrogenase (LDH). Human liver adverse effect for each compound was evaluated as the likelihood of causing elevation in the levels of these enzymes. Chalcone prediction was considered slightly elevated to ALP, SGOT, GGT, as it was also observed for isoniazid and pyrazinamide, highlighting compound **5** for normal level for SGPT. Notwithstanding, good results were found for all chalcones for LDH enzyme, particularly for compound **5** which does not present toxicity for LDH. LDH levels for isoniazid and pyrazinamide were elevated, confirming the hepatotoxic effect of these compounds [23].

### 2.2. Discussion

Chalcones and their derivatives have attracted increasing attention due to numerous pharmacological applications, such as anti-inflammatory and antimicrobial including antimycobacterial effects. Thus, synthetic accessibility of chalcones has generated great interest among organic and medicinal chemists, encouraging experiments aimed at diversification of the core structure of chalcones and the generation of compounds with improved properties [15].

Inflammation is strongly involved in pathogenesis of most infectious diseases, including TB. In general, proinflammatory mediators production by infected macrophages, such as NO and TNF-α, are essential for protection from mycobacteria. TB treatment is based on the use of antimycobacterial drugs. However, many severe forms of TB, such as miliary TB or tuberculous meningitis, require an additional anti-inflammatory therapy to suppress excessive inflammation [24]. For instance, adjunctive treatment of TB with dexamethasone [25] and prednisolone [26] demonstrated a reduction of mortality and a residual neurological deficit in patients with tuberculous meningitis.

An urgent need for effective alternative anti-TB treatment, especially for the severe destructive and disseminated forms of TB frequently associated with exacerbated inflammation, initiated attempts to design and develop compounds with dual activities, *i.e.*, antimycobacterial and anti-inflammatory activities.

In this study, we investigated anti-inflammatory and antimycobacterial effects of a series of forty synthesised chalcones with substituents in A and B rings and one commercially-available unsubstituted chalcone. The identification of most active compounds was accompanied by structure- activity relationship (SAR) analysis. The initial screening of chalcones was performed through evaluation of the inhibitory effects of chalcones on the production of NO and TNF-α by LPS-stimulated cultured macrophages.

More pronounced levels of inhibitory activity against NO were presented by ten chalcones (compounds **4**, **5**, **12**, **24**, **28**, **29**, **31**, **33**, **40** and **41**), exhibiting IC_50_ lower than 21 µM. The most active was compound **5** bearing a methylenedioxy group between carbons 3 and 4 of A ring (IC_50_ 2.1 ± 2.7 µM).

The methylenedioxy group is generally found attached to an aromatic structure where a methylenedioxybenzene or benzodioxole functional group is formed, and it is widely found in natural products. The presence of this group in *Saururus chinensis* lignans [27] and in myristicin, found on *Myristica fragrans* [28] contributed to the capacity of these compounds to inhibit NO production by RAW 264.7 macrophages. To the best of our knowledge, this is the first report demonstrating anti-inflammatory activity for chalcone **5** that has methylenedioxy substituent on the 3,4-position. However, it should be noted that four other compounds evaluated in this study, sharing the methylenedioxy group on ring A, as in compound **5**, and additionally having substituents on ring B, presented lower levels of activity than compound **5**.

Chalcones effectively inhibiting NO were equally effective in suppressing PGE_2_, and these activities were mediated by inhibition of iNOS and COX-2, respectively. In contrast to production of NO and PGE_2_ that was easily suppressed by most chalcones, the production of inflammatory cytokines TNF-α and IL-1β was inhibited by few compounds. Considering that some chalcones exhibited increased cytotoxicity for macrophages and were excluded from the further investigation, only three chalcones (compounds **3**, **4** and **5**) were identified as potent anti-inflammatory agents able to suppress production of principal inflammatory mediators (NO, PGE_2_, TNF-α and IL-1β) by LPS-stimulated macrophages. Compound **3** was characterized by a chlorine group substituted onto the ring A at 4 position and compound **4** presented a 4'-dimethylamino substituent on ring A. Of note, these substituents have been reported in the previous studies as important structural features characteristic for the anti-inflammatory chalcones [29].

In order to identify chalcones with antimycobacterial properties, the chalcone compounds were screened for their ability to inhibit growth of *M. bovis* BCG in culture, and active compounds were further tested against *M. tuberculosis* strains, laboratory strain H37Rv and clinical isolate M299.

Some of chalcones included in this study (compounds **24**, **25** and **30**) have been reported previously to exhibit antimycobacterial activity [16,17,18]. In accordance with these data, compounds **24**, **25** and **30** presented satisfactory anti-BCG activity in our experiments. However, only one of them (compound **25**) was demonstrated to be active against the *M. tuberculosis* M299 strain, two other compounds were excluded from the further tests due to strong cytotoxicity or weak activity against *M. tuberculosis*. It should be noted, that few tested chalcones exhibiting activity against BCG and the laboratory Mtb strain were effective in inhibiting growth of strain M299. Strain M299, belonging to the modern Beijing sublineage of *M. tuberculosis*, induced in mice severe pulmonary immunopathology in mice leading to early death and was characterised in our previous studies as a hypervirulent strain [20]. Only five chalcones (compounds **3**, **4**, **5**, **8** and **25**) showed similar activity profiles against strain M299 and laboratory strain H37Rv. Two chalcones (compounds **15** and **24**) and the standard anti-TB drug rifampicin were significantly less potent against the hypervirulent M299 strain that demonstrated a higher level of resistance of this strain to treatment compared with the strain H37Rv.

Regarding the ability of chalcones to suppress intracellular growth of *M. tuberculosis* in macrophages, three highly active compounds (**4**, **5** and **8**) were selected, highlighting the most active compound **5**. To our knowledge, this compound has never been tested against mycobacteria, however, the antibacterial activity of this compound against *Staphylococcus aureus* and *Clostridium cladosporioides* was demonstrated in one previous work [30]. The antimycobacterial activity against *M. tuberculosis* strain H37Rv of compound **4** was previously reported, although the data obtained in that study did not provide sufficient information about minimum inhibitory concentration (MIC) and selectivity index [19].

A final analysis of obtained data regarding dual biological activities, antimycobacterial and anti-inflammatory, of the studied chalcones, allows the identification of the two most active compounds (chalcones **4** and **5**). Results of the ADME and toxicity *in silico* study demonstrated that all pharmacokinetics parameters of the studied substituted chalcones were found to be within the range of recommended values, and the most active chalcones, such as compound **5**, presented lower levels of predicted hepatotoxicity than the standard anti-TB drugs isoniazid and pyrazinamide.

In conclusion, the obtained data demonstrate that chalcone compounds **4** and **5** are promising agents for further prospective studies aimed at the generation of new anti-TB drugs for the adjunctive therapy of severe TB associated with exacerbated inflammation. This is the first report describing a new approach for the screening of anti-TB chalcones focusing on dual biological activities, such as anti-inflammatory and antimycobacterial. And the first time that these both activities were described for compound **5**.

## 3. Experimental Section

### 3.1. Reagents

Cell culture reagents were purchased from Gibco/Invitrogen (Grand Island, NY, USA). Mycobacteria- specific Middlebrook 7H9 and 7H10 media were obtained from Difco (Detroit, MI, USA); and OADC and ADC supplements were from BD Biosciences (BD, Sparks, MD, USA). Lipopolysaccharide (LPS) from serotype 0111:B4 *Escherichia coli*, N^G^-Monomethyl-l-arginine acetate salt (L-NMMA)—inhibitor of iNOS, (cod. M7033), rifampicin (cod. R7382), indomethacin (cod.I7378), 3-(4,5-dimethylthiazol-2-yl)-2,5-diphenyltetrazolium bromide (MTT) and chalcone (1,3-diphenyl-2-propenone) were purchased from Sigma-Aldrich Co. (St. Louis, MO, USA). The chalcone compounds, rifampicin and indomethacin were dissolved in dimethyl sulfoxide (DMSO, Sigma Aldrich); other reagents indicated for cell treatment were dissolved in sterile phosphate buffered saline (PBS) and sterilized by passage through 0.22 mm nylon filters (Corning Inc., Wilkes-Barre, PA, USA). Anti-iNOS (cod. 610328) and anti-COX-2 antibodies (cod. 610203) were obtained from BD Biosciences (San Diego, CA, USA); and horseradish peroxidase-conjugated secondary antibody was from Santa Cruz Biotechnology (Santa Cruz, CA, USA).

### 3.2. Synthesis of Substituted Chalcones

The synthetic strategy towards the chalcones was based on Claisen-Schimdt reactions between appropriately substituted aromatic aldehydes with acetophenones in absolute ethanol at ambient temperature. The produced chalcones (compounds **2**–**41**) were purified through recrystallization from hot ethanol. Synthesized chalcones and commercially obtained 1,3-diphenyl-2-propenone are depicted in Table 1.

#### Characterization of the Chalcones

The synthesized compounds were characterized by 200 MHz ^1^H- and 50 MHz ^13^C-NMR and mass spectroscopy. Chalcones’ NMR data were in accordance with the corresponding reported literature: **1**, **5**, **28** [30]; **2**, **24**, **25**, **30** [16]; **3**, **6** [31]; **4** [19]; **7** [32]; **8**, **34** [33]; **9** [34]; **10** [35]; **11** [36]; **12** [37]; **13** [38]; **14** [39]; **15**, **39** [40]; **16**, **29** [41]; **17** [42]; **18** [43]; **19** [44]; **20** [45]; **21**, **38** [46]; **22** [47]; **23** [48]; **26** [49]; **27**, **31** [50]; **32** [51]; **33** [52]; **35** [53]; **36** [54]; **40** [55]; **41** [56].

### 3.3. Cell Culture and Treatments

Murine RAW 264.7 macrophage cell line (American Type Culture Collection, Manassas, VA, USA), was cultured in Dulbecco’s modified Eagle’s medium (DMEM-F12) supplemented with 10% Fetal Bovine Serum (FBS) and gentamicin (50 µg/mL) in 5% CO_2_ atmosphere at 37 °C. For experiments, the cells were seeded in 96-well plates (1 × 10^5^ cells/well) and incubated for 24 h at 37 °C. For macrophage stimulation, the cell cultures were treated with LPS (1 µg/mL) and incubated for additional 24 h in the presence or absence of the chalcone samples (samples 1–41) that were used at concentrations of 100, 20, 4 and 0.8 µg/mL. In some experiments, a nitric oxide (NO) inhibitor, L-NMMA (20 μg/mL), was used as a positive control of NO inhibition, and a non-steroidal anti-inflammatory drug indomethacin, known to inhibit production of prostaglandins, was used as a positive control of PGE_2_ inhibition.

### 3.4. Quantification of Proinflammatory Mediators (TNF-α, IL-1β, PGE_2_ and NO)

The frozen culture supernatants samples were thawed and immediately analyzed using commercially available enzyme-linked immunosorbent assay (ELISA) kits to measure TNF-α, IL-1β (BD Biosciences) and PGE_2_ (R&D Systems, Minneapolis, MN, USA), according to the manufacturer’s instructions. Standard curves for each mediator were generated using reference cytokine concentrations supplied by the manufacturer. Nitric oxide (NO) generation was assessed by the Griess method to measure nitrites, which are stable breakdown NO products [57].

### 3.5. Macrophage Cytotoxicity Assay

Cytotoxic effects of chalcone samples on RAW 264.7 cell viability in cultures stimulated with LPS were determined using the LDH (lactate dehydrogenase) release assay as previously described [58]. Cytoplasmic enzyme LDH release into cell culture supernatants was detected using a commercial LDH kit (Doles, GO, Brazil). Cell death was expressed as percentage of LDH release, which was calculated using the following formula: percentage of LDH release = 100 × (test LDH release − spontaneous release)/(maximum release − spontaneous release). The maximum release was determined following dissolution of cell monolayers using 1% (vol/vol) Triton X-100. The spontaneous release was determined in supernatants from cultures incubated in the presence of LPS and DMSO, used as a solvent for chalcone samples. IC_50_ values were calculated by nonlinear regression analysis of log[concentration]/inhibition curves using GraphPad Prism 4 (GraphPad Software Inc., San Diego, La Jolla, CA, USA) applying a sigmoidal dose-response variable slope curve fitting using the different percentage obtained for each corresponding concentration in triplicate and were expressed as means with corresponding 95% confidence interval (CI) from 3 independent experiments.

### 3.6. Detection of iNOS and COX-2 by Western Blot

RAW 264.7 macrophages were plated in Petri dishes (2 × 10^7^ cells·mL^−1^) and incubated for 24 h at 37 °C in 5% CO_2_ atmosphere. After incubation, the confluent macrophage cultures were treated with LPS (1 µg/mL) and chalcone samples with two concentrations of most active chalcones for additional 24 h. The resulted cellular monolayers were washed with PBS 1×, scraped and resuspended in PBS 1×, transferred and centrifuged to obtain cell pellets. The cells were lysed in buffer (10% SDS, 20% glycerol, 5% 2-mercaptoethanol, 2% bromophenol blue and 1 M Tris HCl, pH 6.8, containing protease inhibitors). Protein concentrations were estimated by Bradford method. For western blot analysis, cellular extract samples (60 µg) were submitted to 10% SDS–polyacrylamide gel electrophoresis under non-reducing conditions and transferred to nitrocellulose membranes (Amersham Pharmacia Biotech, Uppsala, Sweden). After blocking with PBS-T (0.5% Tween-20) containing 2.5% (w/v) bovine serum albumin, the membranes were incubated with antibodies against iNOS (1:1000) and COX-2 (1:500) for 1 h at room temperature. After incubation period, the membranes were washed followed by treatment with horseradish peroxidase-conjugated secondary antibody. The resulting membranes were developed using diaminobenzidine/H_2_O_2_ as a substrate for peroxidase. In all electrophoresis experiments, a protein standard ladder (Full Range Rainbow-GE Healthcare, Piscataway, NJ, USA) was used to estimate proteins molecular size. Protein bands densitometric analysis was performed using ImageJ software for Windows (NIH, Bethesda, MD, USA). The value for stimulated condition (LPS- treated cells) was set as 1 and other conditions were recalculated correspondingly to allow ratio comparisons.

### 3.7. Mycobacterial Culture and Evaluation of Bacterial Growth

Three mycobacterial strains differed in virulence were used in this study: avirulent *Mycobacterium bovis* Bacillus Calmette-Guérin (BCG), vaccine strain Moreau, and two *Mycobacterium tuberculosis* strains (low virulent laboratory strain H_37_Rv, ATCC 27294, and highly virulent Mtb Beijing strain M299 isolated from TB patient in Mozambique) evaluated for virulence in previous study [20]. Mycobacterial strains were grown in suspension in 7H9 Middlebrook broth, containing 10% albumin dextrose complex (ADC), 0.5% glycerol and 0.05% Tween-80 at 37 °C under Biosecurity level 3 containment conditions. The suspensions densities were measured by spectrophotometry at 600 nm and corresponding concentrations were determined for each strain serial dilution plating on Middlebrook 7H10 agar plates supplemented with 0.5% glycerol and 10% oleic acid–albumin-dextrose–catalase enrichment (OADC). To study the chalcones’ antimycobacterial activity, we employed the MTT assay to quantify bacterial growth in a liquid medium [16]. The bacterial suspensions were plated (1 × 10^6^ CFU/well in 96-well plate) and incubated in the presence of chalcone samples at concentrations of 100, 20, 4 and 0.8 µg/mL or rifampicin (ranging from 0.0011 to 0.03 µg/mL for *M. bovis* BCG; from 0.00032 to 1 µg/mL for Mtb H37Rv and from 0.008 to 10 µg/mL for clinical Mtb isolate M299). The plates were sealed and incubated at 37 °C and 5% CO_2_ for 7 days for *M. bovis* BCG or 5 days for *M. tuberculosis* strains. After these periods, the bacterial cultures were incubated for 3 h with MTT solution (5 mg/mL) and then treated with lysis buffer (20% w/v SDS/50% dimethylformamide—DMF in distilled water, pH 4.7) for 18 h. Resulted samples optical densities were measured at 570 nm. Untreated bacterial suspensions were used to control spontaneous growth of bacteria.

### 3.8. Infection of Macrophage Cultures and Quantification of Intracellular Growth

RAW 264.7 macrophages were plated (1 × 10^5^ cells/well) in antibiotic-free DMEM-F12 medium supplemented with 10% fetal bovine serum and incubated for 24 h. Prior to infection, mycobacterial suspensions were sonicated for 1 minute to disperse clumps and optical densities were adjusted to 0.1. The macrophage cultures were infected at a MOI of 1:1 (macrophage: bacterium). Phagocytosis was allowed to progress for 3 h. After 3 h, extracellular mycobacteria were removed by washing with PBS 1X and the infected cell monolayers were treated for 4 days with chalcone samples or rifampicin. Macrophage viability was monitored by LDH assay and was over 80% in all experiments. On day 4 after infection, cells were lysed cells with 1% saponin to release intracellular bacteria. Lysate aliquots were diluted 10-fold in PBS, plated in triplicates on 7H10 agar plates and incubated at 37 °C. After 21 days, total CFU were determined.

### 3.9. Molecular Modelling and in Silico ADMET Studies

In an attempt to gain insight on the synthesized compounds’ molecular structure, geometric optimization and conformation analysis has performed using Spartan’10 software. Initially, molecules were subjected to conformational analysis using molecular mechanics calculations (MMFFaq) in order to select the lowest energy conformation. After selecting the best conformer, geometric optimization was performed using the semi-empirical method RM1. Finally, the structures were subjected to single-point calculations using the *ab initio* Hartree-Fock (HF) method with the 6-31G** basis set in order to obtain stereoelectronic and geometric parameters that may be correlated with activity against *M. tuberculosis* H37Rv strain. All chalcones were submitted to *in silico* analysis of ADMET using ADMET Predictor™ (Simulation Plus Inc., Lancaster, CA, USA) as isoniazid and pyrazinamide. This software uses mathematical models, based on quantitative structure-activity relationships (QSAR), to predict molecular descriptors for each studied structure, can therefore estimate a certain property. Pharmacokinetics properties were evaluated through ADMET risk score, which is an indicator for potential ADMET problems that a compound might have, since it provides relevant information about optimization compounds and potential security. Lipophilicity, permeability, ionization, permanent electric charge, and hydrogen bonding are presented in ADMET Risk [59]. Hepatoxicity parameters were predicted with relevant biomarkers: alkaline phosphatase (ALP), serum glutamic oxaloacetic transaminase (SGOT), serum glutamic pyruvic transaminase (SGPT), γ-glutamyl transferase (GGT) and lactate dehydrogenase (LDH). Human liver adverse effect for each compound was evaluated as likelihood in these enzymes levels.

### 3.10. Statistical Analysis

Statistical analysis was performed using variance (ANOVA) one-way analysis and Tukey procedure for multiple range tests, employing Prism 4 software (GraphPad) to assess statistical significance between groups of data. A value of *p* < 0.05 was considered to be significant.
